# Redesigning an Information System that Reduces Health Care Accessibility Effort and Increases User Acceptance and Satisfaction: A Comparative Effectiveness Study

**DOI:** 10.5334/egems.240

**Published:** 2018-10-03

**Authors:** Sandra Long, Karen A. Monsen, David Pieczkiewicz, Julian Wolfson, Saif Khairat

**Affiliations:** 1 Institute for Health Informatics, University of Minnesota, Minneapolis, MN, US; 2 School of Nursing, University of Minnesota, Minneapolis, MN, US; 3 Department of Biostatistics, University of Minnesota, Minneapolis, MN, US; 4 Carolina Health Informatics Program, University of North Carolina, Chapel Hill, NC, US; 5 School of Nursing, University of North Carolina, Chapel Hill, NC, US

**Keywords:** Quality Improvement, Organizational Innovation, Health Services Research, Comparative Effectiveness Research

## Abstract

**Objectives:**

This research tackles a critical issue in modern health care systems—namely, to determine if creating a user-centered health information system that is easy to utilize would lead to consumers who are more satisfied and more likely to accept the system.

**Materials and Methods:**

The health information system is a consumer service center that receives inquiries from consumers on how to find and pay for care. To understand if a system designed to decrease effort results in satisfaction, we redesigned the system, deployed it for 3 months, and then compared consumer satisfaction results to a control group. Satisfaction and Net Promoter surveys were provided to consumers who used the control system and consumers using the redesigned system.

**Results:**

This study was completed over a 6 month continual time period where over 100,000 consumer interactions took place. Using 11 different metrics and data from over 5,000 random system users, it was shown that consumers were more satisfied with an information system designed to reduce their administrative effort.

**Discussion:**

While not all consumer survey results were statistically significant, they all showed a shift towards improved satisfaction with the health care system. Statistically, it was shown that there was a dependency between the design of the system to provide information and many needs of the consumers.

**Conclusion:**

A health care system designed to reduce effort in accessing care results in improved consumer satisfaction. Consumers are also more likely to trust the assistance provided by the organization.

## Introduction

For consumers to successfully improve health outcomes, they must be engaged in, satisfied and trustful that the health care information they receive is accurate in meeting their needs [1,2]. This includes engagement in finding health care providers, deciding on appropriate treatments, and paying for care [1,3,4]. Therefore, to be fully engaged, the consumer must satisfactorily accept the design of the system or process they follow to access information [5]. In a review of literature related to the acceptance and engagement of health information systems, we found the most frequently occurring barriers to include failure of the system to meet consumer needs and consumer lack of trust in the accuracy of information provided by the system [1,6]. The systems consisted of technologies such as web portals, telecommunications, and mobile applications. Since existing solutions that utilize proven methodologies are not meeting the needs of the consumer, it is likely that the requirements were not correctly identified [7].

We considered two primary aspects related to our goal of improved consumer systems acceptance. First, to what degree does the consumer perceive the system to be useful, or “the degree to which a person believes that using a particular system will enhance his or her performance or outcome.” [8] In other words, the consumer trusts that the information received through the system will lead to improved health or wellness. The second aspect is the perceived ease-of-use, or “the degree to which a person believes that using a particular system would be free from effort.” [8] Therefore, we find that minimizing effort of system use for the consumer leads to maximum system design [5,8].

We know that the amount of effort required to find and pay for care differs among consumers dependent upon their diagnosis. Diagnosis determines the number of providers required for adequate care delivery and, as well, affects the likelihood that health insurance will cover or discount the cost of needed services [9]. We find that this likelihood is typically due to the consumer misconstruing the path of treatment aligned with their specific health insurance benefits, or due to obtaining care from a provider who lacks the experience required to adequately treat the specific condition [9].

## Purpose and Aims

The purpose of our study was to determine if consumer acceptance, loyalty, and satisfaction would improve with the implementation of a health information system designed to meet consumer immediate needs in access and payment of care. We hypothesized that if we design a health care system that meets consumer needs by reducing the effort required to access appropriate information this will result in greater trust in the system, a consumer population more likely to engage with the system, and, overall, improved consumer satisfaction. We further surmised that when consumers are satisfied with and have trust in the system they will be more likely to utilize that system for purposes beyond their immediate needs, such as clinical guidance and support, and would be more inclined to recommend the system to other consumers. There is evidence that organizations whose consumers are satisfied with their services and recommend them to potential consumers grow at twice the rate of their competitors [10].

For the purpose of this study, we created a consumer contact center within an established health insurance organization as our health care information system. Consumers interacted with the organization’s representatives through phone and web portals to locate a provider and obtain information regarding payment for services. We used factors such as diagnosis, number of denied claims, and number of inquiries into the health organization to design the system. Consumer satisfaction, distress during the interaction with the system, likelihood to recommend the system, and amount of engagement once the initial inquiry is resolved, were used to measure improvement of the redesigned system.

## Methods

### Study Setting

Consumers make approximately 40,000 contacts to the health organization per month via phone, online chat, and e-mail. Consumer inquiries are both clinical and administrative, and include how to use their health insurance payment plan, where to find care, and what types of treatments to consider based on their diagnosis.

We redesigned the health information system by creating a consumer service center to receive inquiries via phone call or web portal from consumers requiring assistance in locating and paying for care. Our method was to deploy the new system for three months and then compare consumer satisfaction results to a control group. The health information system consisted of representatives, telecommunication technology, desktop tools, and web portals containing secure e-mail and chat. We designed and integrated these components to provide consumers with accurate information to assist in obtaining and paying for care. We then synchronized information across the technologies, adding information if it did not exist, making it consistent across all channels of portal, phone, agent desktop, and mobile app. We updated procedures and workflows and trained health representatives to apply them to assist the consumer to navigate provider visits, and to plan for costs associated with visits and treatments. Representatives coordinated visits with a provider aligned with the consumer’s health needs and insurance plan, and then set up the appointment to decrease effort for the consumer. Our representatives educated the consumer on the cost of various treatment paths related to their diagnosis, the prices charged by the provider, and insurance plan benefits. We identified consumers with diagnoses associated with high risk for misprocessed payments and linked them with health representatives with the ability to immediately adjust claims, avoiding the need to follow up after resolution. This was done in real time in conversation with the consumer so as to decrease frustration with the process. Further, representatives assisted with linking consumers to a clinician to provide treatment decision support. Table 1 summarizes the differences between the control system and the intervention (redesigned system) based on factors related to consumer effort in finding and paying for care [6,11].

**Table 1:** Factors Impacting Effort to Access Health Care and how the System Addressed Them Before and After Redesign.

Factor impacting effort	System Prior to Redesign (Control System)	New system features in Redesign (Intervention System)

**Number People with Claims in Household**	Health care organization (contact center representatives) only addressed issue of consumer they were communicating with	Health care needs were addressed for everyone in the household in one conversation between the caretaker and contact center representatives
**Contact with providers outside of health insurance network**	A list of providers and contact information were given to consumer who was looking for care; consumer then had to contact providers on their own	Representatives provided consumer with providers meeting consumer’s preferences, made sure provider was accepting patients, and set up appointments
**Number of adjustments required on claims**	Consumer had to contact health care organization for adjustments, and navigate between representatives to find one who could make claim adjustment	Consumers with diagnoses where claims are likely to pay incorrectly are directly connected with representatives who have the skill to make adjustments
**Number of claims where payer denied payment**	Consumers were not informed that ancillary procedures such as labs, radiology, and pathology may be covered in a variety of ways by their health care insurance plan	Typical treatment paths and various procedures for diagnoses are explained; Representatives connect consumer with clinical nurses to assist with treatment support
**Number of calls made by consumer**	Consumer often didn’t know additional questions to ask and would need to contact the organization multiple times to get all questions answered	Representative looks up and explains benefits, costs, and accessible providers on each contact; Immediate connection with clinical nurse to assist with treatment support or enrollment in clinical program for ongoing assistance
**Number of Web/Mobile app visits**	Consumer was left to navigate web portal system on their own	Real-time chat was enabled to connect with representatives through web portal; button was added to web and mobile app to directly make a phone call to contact center

### Evaluation Method

Surveys were provided to consumers who used the control system (prior to redesign) and consumers using the redesigned system (intervention). The surveys were administered directly after the conversation was finished by sending it through the same communication channel the consumer used to contact the organization. The surveys were the same in the control and the intervention and consumers were notified they were being recorded. The surveys included questions about how satisfied the consumer was, if they trusted the answer they received, and if the agent they communicated with was helpful. Another method of measurement was through the use of a technology that detects distress in the voice of the consumer [12,13]. It determines when the consumer’s voice implies they are experiencing anxiety or stress. The technology is connected to the call system and applies a linguistic-based psychological behavioral model to the sound and language used in the voice of the consumer to detect distress. The algorithmic model was created from Mattersight Corporation’s analysis of voice data, based on negative behavior patterns of dissatisfied consumers, and follows the guidelines used in previous studies [13,14]. Distress signifies that the consumer is frustrated and putting forth effort to understand resolution [15]. The consumers using the health care information system to find care also have the ability to enroll in clinical health programs to assist them with managing their health. Many consumers are unaware that this is included as part of their health insurance plan. In the redesigned system (intervention), health representatives educated members about this program after helping them find care, and were able to enroll them during the conversation. The number of consumers enrolling in programs was also measured.

Only one of the authors worked with the organization in the system redesign. Other authors acted as evaluators to remove bias that can occur when a person critiques their own work.

### Data Collection and Preparation

The data for the consumers served by the health insurance organization is stored and maintained within the organization. This study was conducted within the organization so that HIPAA regulations were followed and both personal health information and personally identified information were secure and not shared outside of agreements in place between the organizations and the consumers in the study. The organization approved this study and consultation took place with its legal department. The information related to factors contributing to effort was spread over multiple servers used to process consumer interactions. Payer entities (employer, government, and individuals) provided consumer identification data to the health care organization. This included demographics such as age, location, race, and sex. Data related to diagnosis was pulled from medical claims submitted by providers which contain ICD codes. The operational processing of these claims is also securely stored so that frequency of payment difficulties could be obtained. These medical claims dataalso shows the cost of treatments. The health care organization stores historical documentation of consumer inquiries for continual use in improving service to consumers. The information related to the defined factors was moved into a single database to act as a place to conduct analyses and inform the health representatives about the specific consumer. This allowed information to easily be moved into Excel, SAS, and Minitab as needed for further data analysis.

Two types of surveys are regularly administered to the consumers to understand how satisfied they are with the system. One is a general satisfaction survey consisting of 8 questions and using a scale of 1 to 5. The other is a Net Promoter Score survey asking 1 question about likelihood to recommend the service. It uses a scale of 1 to 10. They are sent in both written and voice form depending on the communication channel the consumer used for their inquiry. This survey data was collected for both the control and treatment groups and matched to the rest of the consumer data based on the consumer’s health insurance ID number.

The general satisfaction survey was administered directly after the interaction with the system. It was randomly delivered to 10 percent of those contacting the organization, which is shown in Table 2.

**Table 2:** Survey Questions to Understand Satisfaction with the System.

Survey questions administered to consumers after they interact with the health information system	

1.	Thinking about the conversation you had with our agent, overall, **how satisfied** are you with the level of service you received? Remember, the scale of 1 to 5, where 1 means not at all satisfied and 5 means completely satisfied.
2.	How satisfied are you with the agent being **polite and respectful** to you during the call?
3.	How satisfied are you that the agent **understood your issue**?
4.	How satisfied are you with the level **responsibility the agent took** to answer your question or to resolve your issue?
5.	How satisfied are you with the agent’s ability to work through and **resolve your issue**, using **language you understand**?
6.	Using the same rating of 1 to 5, please rate the **trust** you have in the answer you received? 5 means that you have full trust in the answer you received, 1 means you have no trust in the answer you received.
7.	Using a scale of 1 to 5 where 1 means “very high effort on your part” and 5 means “very low effort”, thinking of your most recent call center experience, how much **effort** did you personally have to put forth to handle your request?
8.	Using a scale of 1 to 5 where 1 means “highly disagree” and 5 means “highly agree”, did your customer service representative demonstrate **concern for your needs**?

The Net Promoter Score survey given to consumers consisted of one question, “How likely are you to recommend the service to others?” This survey is randomly sent through phone calls or e-mail to consumers who have the health insurance plan, whether they interact with the information system or not. In this survey, consumers are able to rate the service on a scale of 1 to 10, with 10 being the most likely to recommend. When asking consumers this question, it is considered to be a way to calculate a Net Promoter Score, and was developed as a measurement tool to align consumer satisfaction and loyalty to business profits [10]. The score is calculated by taking the percent of consumers promoting the service (ranking it as 9 or 10) and subtracting out the percent of detractors (those rating it 6 or less) [10]. This survey was administered to random consumers who utilize the organization’s health insurance plans. The consumers did not necessarily need to interact with the health information system.

Throughout the study, other data was collected for random interactions with consumers. This included what was discussed during the interaction, length of interaction time, and behavior of the consumer. For example, whether or not the consumer’s voice sounded distressed was recorded, as well as whether or not the consumer enrolled in a clinical health program during the conversation.

### Data Analysis

A comparative-effectiveness study approach was utilized; the survey results from those using the newly designed system was compared to the results of the control system. The control group was those consumers looking for similar health care services, with the same type of insurance plan, who utilized the system prior to the redesign. To ensure that the minor plan changes throughout the year were not driving the results, the likelihood of consumers to recommend the health care organization for those interacting with the system were compared to those with the same insurance plan who obtained care on their own without assistance from the system.

To validate the survey data, the percent of callers who were distressed according to the voice detection technology in the control was compared to the percent of callers who were distressed when using the redesigned system (intervention). A comparison between control and redesign (intervention) was also conducted for the percent of consumers who enrolled in a clinical program during the conversation.

Chi-square analyses and descriptive statistics were then used for the comparison of the control and the redesign (intervention). This was done since the populations were not exactly the same size, the consumers surveyed before and after the redesign consisted of different individuals, and the distribution of the results was not normal.

## Results

This study was completed over a 6 month continual time period where over 100,000 consumer interactions took place. Using 11 different metrics and data from over 5,000 random system users, it was shown that consumers are more satisfied with a health information system designed to reduce their administrative effort. This was shown through 9 survey questions, level of distress in the voice of the consumer, and the likelihood that a consumer would accept clinical assistance from the organization.

The general satisfaction surveys that were administered directly after the interaction between consumers and the health information system used the Likert scale of 1 through 5, with 1 being the least desired response and 5 being the most. While the distribution of all of the survey data was weighted more towards a 5, with the median always being 5, a shift of the population scoring higher was observed. This means that less people rated the system as 1 or 2 and more consumers rated it as a 4 or 5. Table 3 shows the results from the surveys. The pattern of consumers shifting to more desired responses was observed for each question and results are shown in the table with shaded and bold text. The results of each chi-square test are also shown. Questions 3, 5, 6, and 7 showed a statistically significant difference with a p-value of less than 0.05. This means that consumers did feel the agent better understood their issue, resolved it in a way they understood, that they had more trust in the answer received, and put forth less effort with the intervention (redesigned system). Even though the other 4 questions did not show a statistically different result, a pattern was observed showing a shift to greater satisfaction.

**Table 3:** Survey Results from Questions Asked Immediately After Interaction with the System.

		1. How Satisfied		2. Polite And Respectful		3. Understood Your Issue		4. Responsibility The Agent Took	

Survey		*p = 0.309*		*p = 0.608*		*p = 0.034*		*p = 0.086*	

Result		Control	Redesign	Control	Redesign	Control	Redesign	Control	Redesign

1	n	**186**	184	**98**	90	**148**	137	**157**	153
	*%*	***4.057***	*3.692*	***2.137***	*1.806*	***3.228***	*2.749*	***3.424***	*3.07*
2	n	**90**	74	**41**	39	**85**	60	**81**	63
	*%*	***1.963***	*1.485*	***0.894***	*0.783*	***1.854***	*1.204*	***1.767***	*1.264*
3	n	143	146	72	69	116	112	110	126
	*%*	*3.119*	*2.929*	*1.57*	*1.384*	*2.53*	*2.247*	*2.399*	*2.528*
4	n	377	**428**	176	181	277	**325**	268	**336**
	*%*	*8.222*	***8.587***	*3.839*	*3.632*	*6.041*	***6.521***	*5.845*	***6.742***
5	n	3789	**4152**	4198	**4605**	3959	**4350**	3969	4306
	*%*	*82.639*	***83.307***	*91.559*	***92.396***	*86.347*	***87.279***	*86.565*	*86.396*
		**5. Resolve Your Issue**		**6. Trust In Answer Received**		**7. Effort Put Forth**		**8. Concern For Your Needs**	

**Survey**		***p = 0.041***		***p = 0.029***		***p = 0.001***		***p = 0.546***	

**Result**		**Control**	**Redesign**	**Control**	**Redesign**	**Control**	**Redesign**	**Control**	**Redesign**
1	n	**167**	160	**202**	190	**307**	244	**169**	169
	*%*	***3.642***	*3.21*	***4.41***	*3.81*	***6.7***	*4.9*	***3.686***	*3.391*
2	n	**84**	68	**101**	87	**153**	144	**72**	68
	*%*	***1.832***	*1.364*	***2.2***	*1.75*	***3.34***	*2.89*	***1.57***	*1.364*
3	n	126	104	222	200	329	325	153	145
	*%*	*2.748*	*2.087*	*4.84*	*4.01*	*7.18*	*6.52*	*3.337*	*2.909*
4	n	300	**348**	564	585	667	**744**	347	**392**
	*%*	*6.543*	***6.982***	*12.3*	*11.74*	*14.55*	***14.93***	*7.568*	***7.865***
5	n	3908	**4304**	3496	**3922**	3129	**3527**	3844	**4210**
	*%*	*85.234*	***86.356***	*76.25*	***78.69***	*68.24*	***70.77***	*83.839*	***84.47***

**Figure 1:**
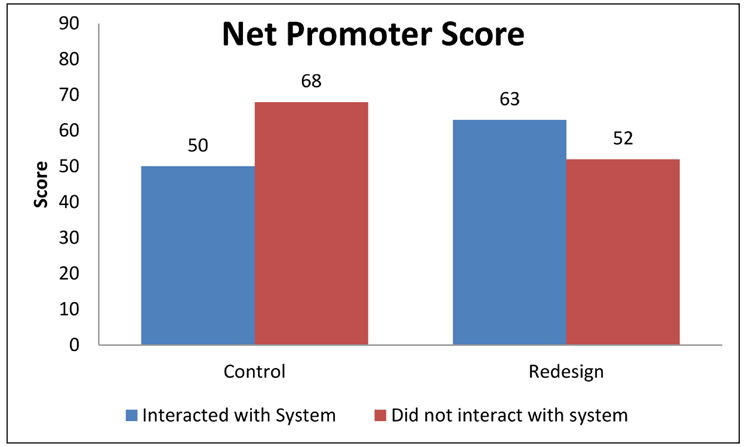
Net Promoter Scores from Consumers with the Same Health Insurance Plan.

The results from the Net Promoter survey about likelihood to recommend the service to others showed improved scores for consumers interacting with the system. Consumers who did not interact with the system, but utilized the same health insurance plan, scored lower for the same time period. In this example the control was considered to be consumers with organization administered health insurance plans in the time period prior to the redesign (intervention), whether they interacted with the system or not (n = 134). The “Redesign” population consisted of consumers with the same plan after the new system was implemented (n = 196). The score for those interacting with the system increased by 13 points and those who did not interact with the system had a score that decreased by 16 points. Figure 1 shows the results from the survey.

**Figure 2:**
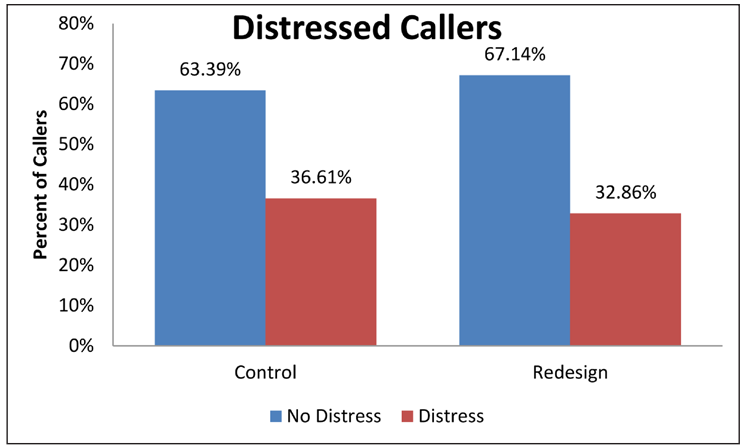
Percent of Callers in Control and Redesign Whose Voice Indicated Distress.

After analyzing the percentage of callers into the organization that were distressed, the redesigned system (intervention) had approximately 4 percent less consumers whose voice sounded distressed out of the entire population. This resulted in close to a 12 percent reduction in distressed callers overall. The sample size for the control was 10,259 calls and the sample size for the redesign (intervention) was 8,938 calls. The chi-square test had a significant p-value of less than 0.001, showing a dependency between the system design and distressed callers. Figure 2 shows the results from the distressed caller study.

When the data related to program enrollments was analyzed, it was discovered that conducting comparison tests was challenging due to health insurance payers offering different types of clinical programs. Some plans only offered programs assisting consumers experiencing cancer while others offered programs to assist with general health and wellness, such as exercise and diet coaching. Another issue was due to the fact that outbound campaigns to get consumers to enroll in the programs were taking place at the same time as the testing for this study. It is possible that the redesigned system (intervention) had more enrollments due to a program marketing campaign being sent out at the same time as the redesigned system implementation. Due to this, two health insurance plan populations with the same types of programs and campaigns needed to be compared. One population was consumers with a plan that did not have the redesigned system implemented (control), and the other had consumers who could interact with the new system. The population that had the ability to interact with the new system had 19 percent more enrollments in clinical programs than the control.

## Discussion

While not all results of the survey questions asked to consumers were statistically significant, they did all show a shift towards improved satisfaction with the health care system. Consumers appeared to be more satisfied with the health care organization, felt the system was more concerned about their needs, and that it better understood their issues. Statistically, it was shown that there was a dependency between the design of the system to provide information and many needs of the consumers. In the redesigned interface, consumers were more likely to feel the health representatives understood their issue, information was communicated in a way that was understood, they had trust in the answer, and there was low effort on the part of the consumer themselves. This lower effort with the redesigned system (intervention) implies that the consumer is more likely to accept the system as one assisting them with finding and paying for health care [8,16].

Consumers are not likely to recommend an organization to their friends and acquaintances if they are unsatisfied with it. Research has shown that the more likely consumers are to recommend a system contributes to increased growth and profits for an organization [10]. While there is literature that aims to show reduced medical costs, there is little that directly shows how revenue and growth can be obtained through improved health information systems. This study showed that consumers are more likely to recommend the system when it is designed to reduce their effort to find and pay for health care. The consumers using the redesigned system (intervention) promoted the system more than those using the control system.

Frustration and effort to find resolution can come across in the voice of a person [17]. In the study, the distress detected in the voice of a consumer was reduced with the redesign of the system. While studies show success using distressed voice detection technology, this is the first showing it with a health information system [13]. It is a better use of consumer’s time to focus on administering the treatments prescribed and activities directly contributing to their health rather than the stress of finding and paying for the care [18]. This study showed that stress was reduced in the population utilizing the system redesigned to reduce effort in accessing care. People undergoing recovery from ailments are more likely to achieve improved health when they have lower stress [18].

Many health information system studies aim to show acceptance and satisfaction with a system using improved health outcomes as a measure [1]. This study differs in that it focuses upstream before a consumer undergoes their full treatment plan to build satisfaction and engagement through administrative tasks. This then sets the system up to provide additional clinical related services.

Consumers were more likely to seek assistance from the health care organization when they also had a service model to reduce the effort they had to put forth in accessing care. Within the organization studied there are clinicians who provide treatment decision support to consumers. Consumers did not utilize or engage with clinicians to the same degree prior to the redesign as they did once the system met their needs in finding and paying for care. This ties to literature related to trust and may be due to the fact that consumers felt they received correct answers with their original inquiries, so they were more willing to trust the organization for additional services [1,3].

The new system was implemented at the organizational scale across the United States. The cost of implementation and training of the agents was covered by the improved and more affordable health care value that resulted from engaging more consumers in clinical programs. Therefore, satisfaction in using the system increased without an overall cost increase to the organization. The implementation consisted of providing agents with online modules and instructor led training on the new procedures and workflows.

### Limitations

One limitation of this study was that it was not feasible to ask the same consumer the survey questions with both the control and redesigned system (intervention). This is due to the fact that consumers have different health needs throughout their lives and over the time of the study. Another limitation is that consumers answering their questions may have been at different points of the treatment plans. The large sample size assisted with overcoming this limitation, but future studies could be done to provide a better comparison. The distribution of Likert scale data was weighted more toward high satisfaction so non-parametric analyses needed to be used. The sample sizes between control and treatment were close in size, but different since consumers were not required to complete the surveys. Additional studies that could be conducted to test satisfaction in finding and paying for care could take place with digitally designed systems. Lower effort payment processes and provider searches on mobile apps and websites could be designed to increase satisfaction and acceptance.

## Conclusion

A health care system designed to reduce effort in accessing care results in improved consumer satisfaction. Consumers are also more likely to trust the assistance provided by the organization. Finding and paying for care must happen for consumers to see clinicians and obtain the prescriptions required to improve their health conditions. Easier access to health care allows consumers to find it sooner and focus on engaging with providers. When consumers have assistance that reduces effort in managing medical claims and finding providers, they are more likely to accept the health care system.
